# Reproductive Cycle of *Marphysa sanguinea* (Montagu, 1815) (Polychaeta: Eunicidae) in the Lagoon of Tunis

**DOI:** 10.1155/2013/624197

**Published:** 2013-06-04

**Authors:** Monia El Barhoumi, Patrick Scaps, Fathia Zghal

**Affiliations:** ^1^Laboratoire de Biologie de la Reproduction et du Développement Animal, Faculté des Sciences de Tunis, Campus Universitaire, El Manar 2092 Tunis, Tunisia; ^2^Laboratoire de Biologie Animale, Université des Sciences et Technologies de Lille, 59655 Villeneuve d'Ascq Cédex, France

## Abstract

The reproductive cycle of *Marphysa sanguinea* (Polychaeta: Eunicidae) was studied in the Lagoon of Tunis between May 2006 and May 2007. *M. sanguinea* is a gonochoric species. There were no morphological differences between males and females, and spawning occurred without epitokal metamorphosis. Gonads are not well defined in either sex. The process of spermatogenesis takes place in the coelomic cavity. Mature males show all stages of spermiogenesis at any one time. The ovaries of *M. sanguinea* consist of coelomic germ-cell clusters surrounded by a thin envelope of follicle cells derived from the peritoneum. Germ cells in premeiotic and previtellogenic phases are observed in one cluster. In each cluster the more differentiated oocytes detach and float free in the coelomic cavity where they undergo vitellogenesis as solitary cells. The cytoplasmic material of the mature oocytes (diameter superior to 200 **μ**m) is asymmetrically distributed; large lipid droplets and large yolk spheres occupy the vegetal pole of the oocyte while smaller yolk spheres are situated in the animal hemisphere. The female coelomic puncture has a heterogeneous aspect and shows different oocyte diameters. The reproductive period is more intense in winter period from January to March. Spawning occurs mainly in April.

## 1. Introduction

Polychaetes display an extraordinary diversity of reproductive traits [1], probably due to their high plasticity and adaptability to different habitats [2–7]. Polychaetes, located in unpredictable environments, have been deeply studied to understand the characteristics of their life cycle. Fauchald [8] has divided polychaetes into three general reproductive life styles. Later, Wilson [4] described a two-factor classification system for types of reproductive modes within the Polychaeta based on the type of larval development and the fate of the female gametes (free spawned or brooded in a variety of ways). Mettam [9], Olive [10], and Prevedelli and Cassai [11] related this diversity of reproductive traits to the importance of variation in life history traits related to the characteristics of brackish environments, presence or absence of epitoky, and reduction or disappearance of the dispersal phase.

The polychaete *Marphysa sanguinea* (Eunicidae) was briefly described by Montagu in 1815 [12] from the south coast of England. This species is recorded as a cosmopolitan species, distributed globally at temperate to tropical latitudes (e.g., [13–15]). However, voucher specimens for many populations do not exist or were poorly identified. Taxonomists have recently reexamined many of these specimens, concluding that they may be a few to several different sibling species [15]. The presence of *M. sanguinea* on the coast of Tunisia has been first reported by Ben Amor in 1984 [16] from Zembra island. This species is also present in the Lagoon of Tunis where it is used as bait for sport and commercial fishing. It is one of the most important economic resources of the lagoon. Unfortunately, no studies have targeted the reproductive biology of the species from Tunisian waters. Moreover, only fragmentary data are available on reproductive biology of *M. sanguinea *from natural populations [15]. In this study we report for the first time some aspects of the reproductive biology of *M. sanguinea* from Tunisian waters. 

## 2. Materials and Methods

**Figure 1:**
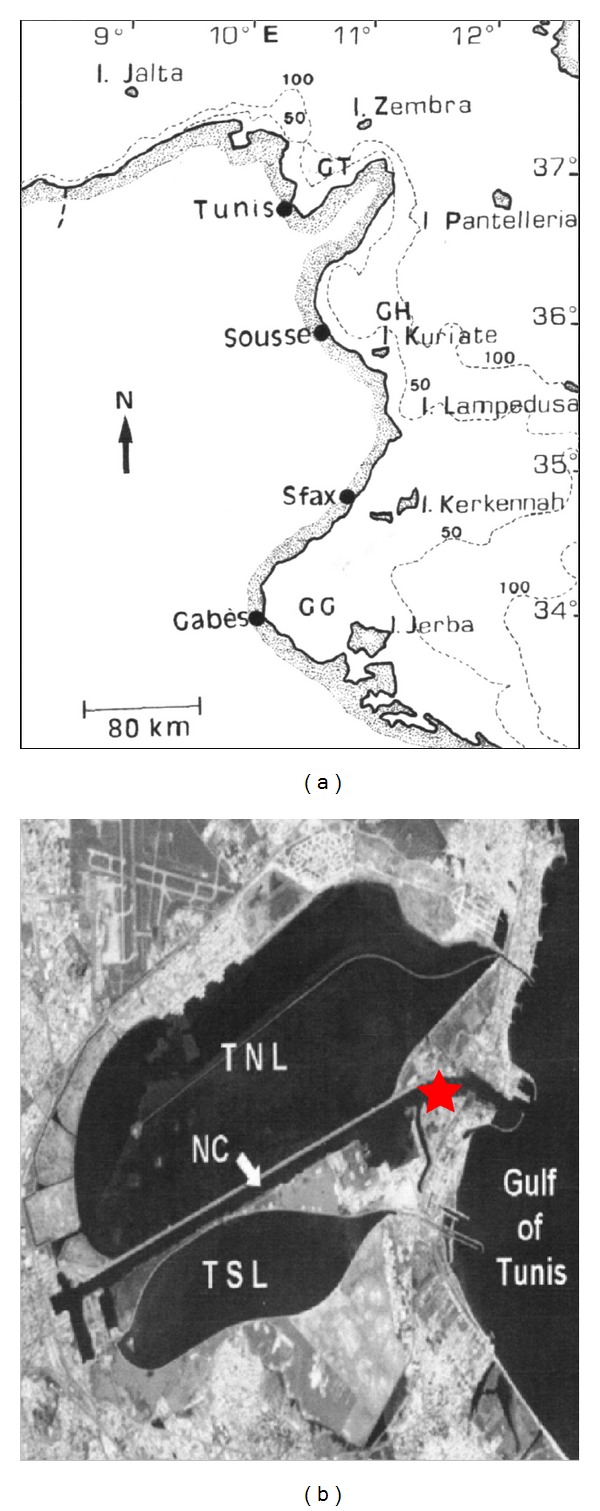
(a) Map of Tunisia with the Gulf of Tunis (GT), the Gulf of Hammamet (GH), and the Gulf of Gabes (GB); (b) Map of Tunis Lagoon divided by channel navigation (NC) in two areas, Tunis Northern Lagoon (TNL) and Tunis Southern Lagoon (TSL), and location of the sampling site inside the navigation channel.

The Lagoon of Tunis, adjoining the city of Tunis, is located in the southwestern Gulf of Tunis (Figures 1(a) and 1(b)). It is a Mediterranean eutrophic coastal lagoon covering 45 km² to an average depth of 1 m. It is characterized by high fluctuations of physicochemical conditions. It is divided in two areas by a navigation channel. Individuals of *Marphysa sanguinea* (Montagu, 1815) were collected monthly in the navigation channel (36°48.452′ N; 10°18. 321′ E) from May 2006 to May 2007. *M. sanguinea* is one of the most common species of errant Polychaeta in the Lagoon of Tunis. *Marphysa* is a burrowing polychaete found in sandy and muddy environments or under stones. It attains a maximum length of about 40 mm. The individuals occur low in the intertidal zone and extend down into the sublittoral; in consequence, individuals were collected in the intertidal zone. Reproductive characteristics were analyzed in all the individuals collected in an area of 2 m² dug to a sediment depth of about 50 cm. After collection, individuals were rinsed with filtered seawater and maintained individually in covered containers filled with about 100 mL of filtered seawater. In the laboratory, a small quantity of very fine sand was added to containers. The containers were stored at 5°C before utilization.

Sexual maturity was determined by examination of histological sections and light microscopic observation of the fresh coelomic fluid. Individuals were fixed in alcoholic Bouin's fluid, dehydrated, and prepared for conventional paraffin wax microscopy. After dehydration through ethanol series (70%, 95%, and 100%) and storage in butylic alcohol, the fixed material was embedded in paraffin. Wax sections were cut at 5–7 *μ*m and stained with hematoxylin-eosin technique. As external sex differences are lacking in adults, the sex of each individual was determined after examination of coelomic punctures. The diameter of at least 100 oocytes was measured using a calibrated eye piece graticule. Males were recognized by the presence of clusters of germ cells and mature ones by the presence of sperm. Those animals without sexual products were considered to have an undetermined sex.

## 3. Results

### 3.1. Sex Ratio

The reproductive characteristics were analyzed for a total of 40 to 65 individuals per month. *Marphysa sanguinea* is a gonochoric species, with individuals being either male or female. There were no morphological difference between males and females, but the latter could be distinguished for part of the year by the presence of oocytes, visible through the body wall in the coelomic cavity.

**Figure 2:**
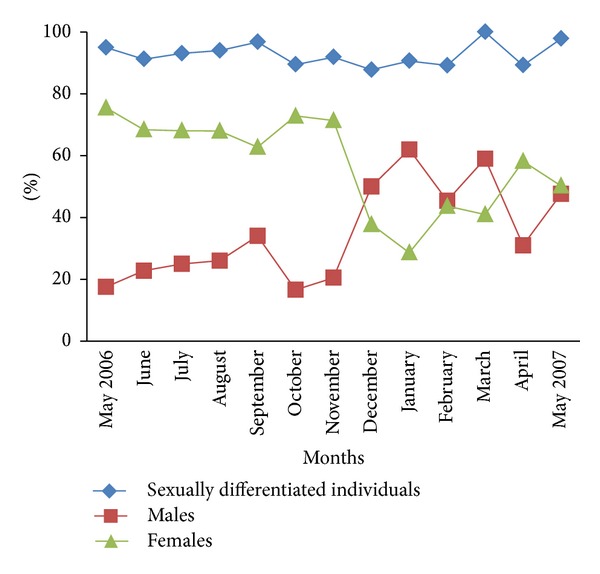
Monthly proportions of sexually differentiated individuals, females, and males from May 2006 to May 2007.

Epitokous or schizogenic metamorphosis has never been observed in this population. Throughout the investigation, 389 specimens were female, 189 male, and 48 undetermined. In all monthly samples, undetermined individuals were very few and were represented only by juveniles that still had to start, or had just begun, the gametogenesis processes; so the proportion of sexually differentiated individuals was high and constant (*≈*90%) throughout the sampling period (Figure 2). In contrast, the proportions of males and females fluctuated greatly. The ratio of females within the population was higher (between 60 and 80%) than that of males (between 20 and 30%) from May 2006 to November 2006; this is probably due to the fact that this period corresponds to the moment when the reproduction is less intense and that it was more difficult to recognize clusters of spermatogonia than to recognize small oocytes. In December we noted a decrease in the proportion of females and an increase in the proportion of males. The proportion of males was maximal (between 40 and 65%) from December to March due to the differentiation and maturation of males while the proportion of females was minimal during the same period (between 30 and 40%) reflecting the maturation of females. In April and May the sex ratio was close to 1 : 1.

### 3.2. Male Sexual Cycle

**Figure 3:**
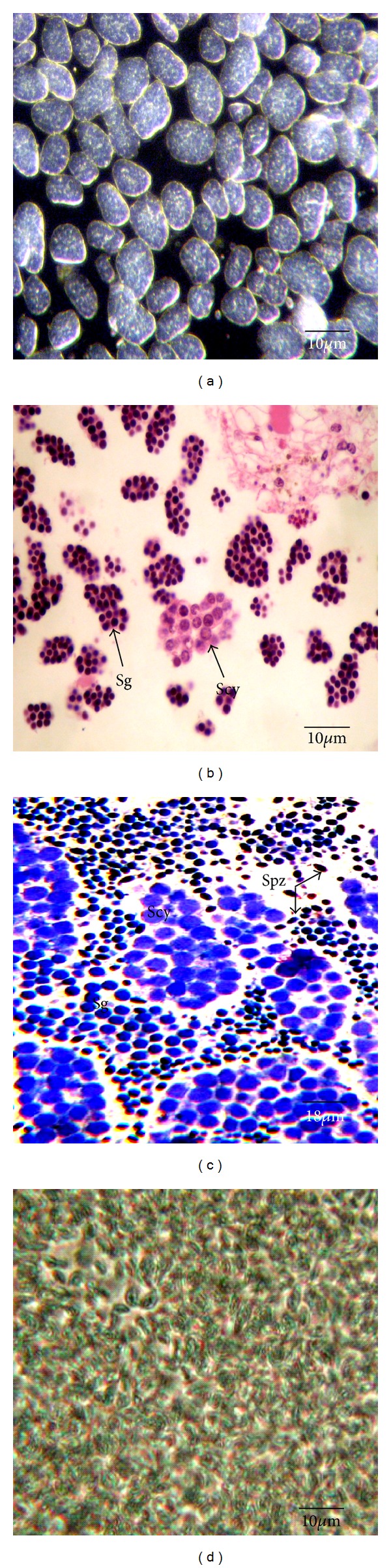
Spermatogenesis. (a) Coelomic fluid from a male collected in June showing early germ cells arranged as mulberry-like clusters; (b) clusters of spermatogonia (Sg) and primary spermatocytes (Scy) in the coelomic fluid of a male collected in July; (c) coelomic fluid from a male collected in October containing sperm in all stages of development: spermatogonia (sg), primary spermatocytes (Scy), and spermatozoa (Spz); (d) coelomic fluid of a mature male collected in December showing free sperm.

Testes were not observed in *M. sanguinea*. The process of spermatogenesis took place in the coelomic cavity. The germinal elements were released from the peritoneum into the coelomic cavity as mulberry-like clusters of spermatogonia (Figure 3(a)) and all the following divisions occurred in the coelom (Figures 3(b) and 3(c)). The spermatogenesis was not synchronous and all stages of spermatogenesis could be found at any time in the coelom of males (Figure 3(c)). Nevertheless, the frequency of the different stages of the spermatogenesis varied according to the season. Males were considered mature when the proportion of free spermatozoa was high and the proportion of clusters of spermatogonia was negligible. The percentage of mature males was maximal from December to April (between 40 and 93%) indicating that this period corresponded to the most intense reproductive period. A few isolated mature males were found from June to September (between 20 and 37%) and in November (28%) while no mature males were observed in May and October indicating the presence of a sporadic reproduction.

### 3.3. Female Sexual Cycle

The examination of histological sections and observation of sexual products allowed us to describe the morphological oocytes aspects at different stages. 

**Figure 4:**
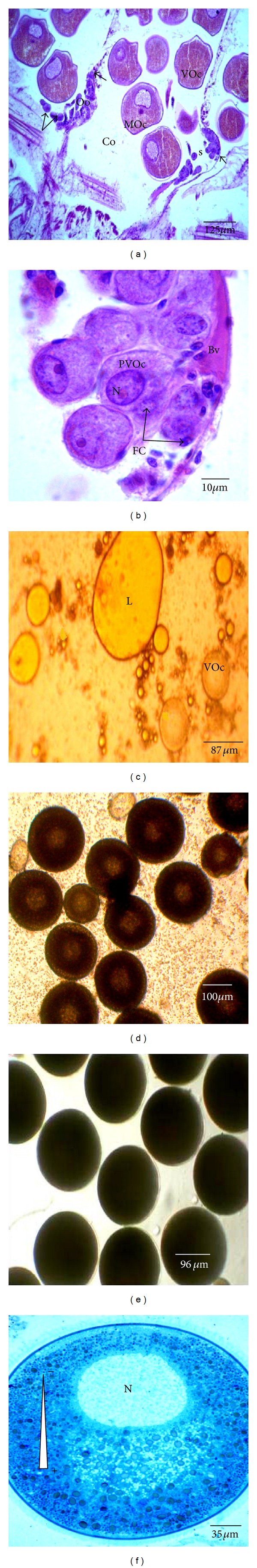
Ovogenesis. (a) Histological section of a posterior segment of a female collected in March showing ovaries (arrowheads) containing primary oocytes (arrows), oogonia (Oo), free vitellogenic (VOc) and mature oocytes (MOc) in the coelom (Co). (b) Detail of an ovary from an individual collected in May. The ovary consists of a germ-cell cluster surrounded by a thin envelope of follicle cells (FC) (arrows) and is attached to a blood vessel (Bv). (c) Coelomic fluid (Co) from a female collected in August showing early vitellogenic oocytes (VOc) and numerous lipid droplets (L). (d) Coelomic fluid from a female collected in September showing vitellogenic oocytes. (e) Coelomic fluid from a female collected in December showing mature oocytes. (f) Detail of a mature oocyte from a female collected in January. The arrow indicates the gradient of distribution of the vitellus. N: nucleus; PVOc: previtellogenic oocyte; S: germinal sac.

The ovaries of *M. sanguinea* were discrete organs present in the posterior segments and consisted of coelomic germ-cell clusters surrounded by a thin envelope of follicle cells (i.e., sheath cells according to Fisher, 1975) derived from the peritoneum (Figures 4(a) and 4(b)). Clusters were attached to the genital blood vessels (Figure 4(b)). Females usually had two clusters per segment except for the first segments. Oogenesis was of the extraovarian type. Germ cells in premeiotic and previtellogenic phases were observed in one cluster (Figure 4(b)). In each cluster the more differentiated oocytes detached from the ovary and completed vitellogenesis while floating freely in the coelomic fluid (Figure 4(a)). Vitellogenesis took place entirely outside the ovary. 

Oogenesis consisted of a proliferative phase followed by a prolonged growth phase resulting in the production of mature oocytes. During the proliferative stage oogonia and young oocytes were small (8 *μ*m in diameter). Prior to the beginning of vitellogenesis, the oocytes increased in diameter. They began to elongate and measured about 25–35 *μ*m in length. Late previtellogenic or early vitellogenic oocytes broke free from the clusters and float free in the coelomic cavity where they underwent yolk synthesis as solitary cells. Vitellogenesis resulted in a rapid increase in the volume of the oocyte largely due to the accumulation of nutritive material in the ooplasm (Figure 4(d)). During the vitellogenic phase numerous lipid droplets were seen in the coelomic cavity (Figure 4(c)). 

Mature oocytes (Figure 4(e)) (diameter superior to 200 *μ*m) were characterized by a large eccentric nucleus (Figure 4(f)). Cytoplasmic material was unevenly and asymmetrically distributed in the mature oocytes. Large lipid droplets and large yolk spheres occupied the vegetal pole of the oocyte while smaller yolk spheres were situated in the animal hemisphere (Figure 4(f)). 

The female coelomic puncture had a heterogeneous aspect and showed different oocyte diameters. Due to the presence of previtellogenic, vitellogenic, and sometimes mature oocytes in the coelom of females we deduced that *M. sanguinea* had an asynchronous oogenesis. For this reason, a biometric study of oocytes growth was essential to determine the reproduction period as well as the spawning season.

**Figure 5:**
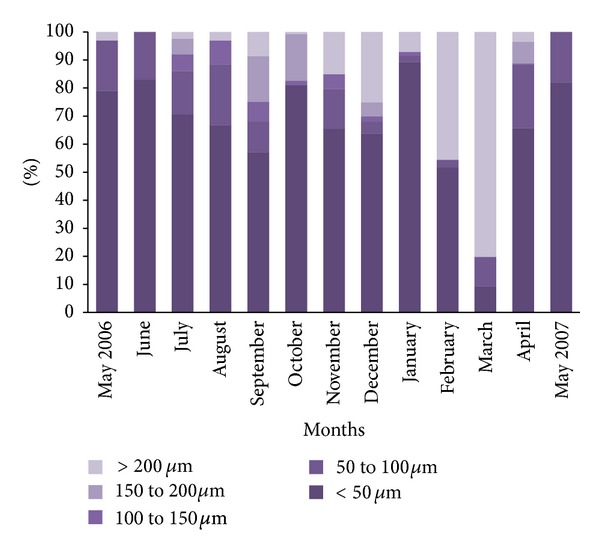
Size-frequency distribution of oocytes from May 2006 to May 2007.

The diameter of the oocytes present in the coelomic cavity was used as indicator of the maturation stages. In *M. sanguinea* these germinal elements had a wide range of dimensions, so five groups of increasing oocytes were identified using a class interval size of 50 *μ*m (Figure 5). Oocytes that had completed vitellogenesis measured 280–300 *μ*m. Females with small oocytes (diameter less than 50 *μ*m) were present throughout the year (Figure 5).

**Figure 6:**
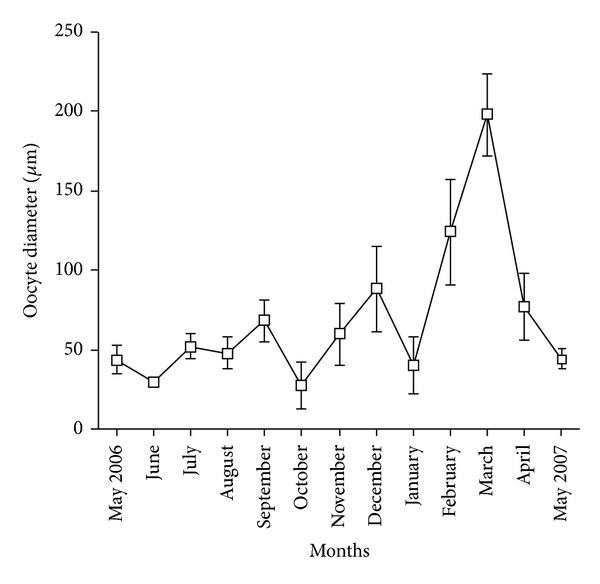
Evolution of oocyte size from May 2006 to May 2007. Each data represents mean ± standard error of the mean (SEM).

The evolution of mean oocyte diameter for females during the study period (Figure 6) allowed us to reveal the female sexual cycle. The mean oocyte diameter varied between 27.63 ± 14.50 *μ*m in October and 197.74 ± 25.55 *μ*m in March. From April to January, the mean oocyte diameter ranged between 20 and 80 *μ*m. We recorded a steady increase of the mean oocyte diameter from January to March with a mean maximal value of 197.74 ± 25.55. The coelomic fluid of females collected in March contained a majority of large oocytes (Figure 5) indicating that this period corresponded to the most intense reproductive period. From April the drastic reduction in the number of mature oocytes in the coelomic cavity was connected with spawning. The phase of increase of the mean oocyte diameter was quite synchronous with spermatogenesis phase.

A relatively high proportion of females containing mature oocytes was found from November to January (between 9 and 25%) and a few isolated mature females were found in April and from July to September (Figure 5) indicating the presence of a sporadic reproduction. During spring and summer periods we found a high proportion of females containing small and medium oocytes (Figure 5). 

## 4. Discussion

*M. sanguinea* is a gonochoric species, with individuals being either male or female. Gonads are not well defined in either sex. Gametogenesis is of an extragonadian type. *M. sanguinea* has asynchronous spermatogenesis and oogenesis The ovaries of *M. sanguinea* are discrete and consist of coelomic germ-cell clusters surrounded by a thin envelope of follicle cells derived from the peritoneum. Late previtellogenic oocytes detach from the clusters and float free in the coelomic cavity where they undergo vitellogenesis as solitary cells. Oocytes that had completed vitellogenesis measure 280–300 *μ*m. The same diameter of mature oocytes was measured in females collected in the Venice Lagoon [15]. The cytoplasmic material of the mature oocytes is asymmetrically distributed; large lipid droplets and large yolk spheres occupy the vegetal pole of the oocyte while smaller yolk spheres are situated in the animal hemisphere. The same type of eggs was observed in many other species of polychaetes such as *Nereis*, *Platynereis,* and *Diopatra* [17, 18]. It can be hypothesized that the differential distribution of cytoplasmic components leads to qualitative differences in blastomere cytoplasm. Elsewhere it has commonly been thought that these differences are responsible for the process of cell diversification and embryonic axis formation in early stages of embryonic development [19]. 

As for the majority of polychaetes [20], the male germ cells in *M. sanguinea* are released from the peritoneum into the coelomic cavity in mulberry-like clusters at an early stage of spermatogenesis.

Contrary to what occurs in many Eunicidae [21, 22] we do not observe modifications characteristics of epitoky or shizogamy in the adults of *Marphysa sanguinea* from the Lagoon of Tunis. Epitokous metamorphosis was not observed too in another population of *M. sanguinea* from the Venice Lagoon [15]. According to Prevedelli et al. [15], the disappearance of the epitokal phase in species that colonize brackish habitats seems to be generalized. The suppression of epitoky is a common trend in estuarial species [9–11] and is probably related to a reduction in the dispersal phase [7]; species living in unpredictable habitats such as brackish environments tend to limit spatially the gametes emission.

*M. sanguinea* displays a seasonal and synchronous reproduction in the Lagoon of Tunis. We recorded a steady increase of the mean oocyte diameter from January to March indicating that this period corresponded to the most intense reproductive period of reproduction. However, we also found a relatively high proportion of females containing mature oocytes from November to January; so, we can consider that the reproductive period extends from November to March. The percentage of mature males was maximal from December to April. So, ovogenesis and spermatogenesis phases were quite synchronous. The reproductive cycle of *M. sanguinea* in the Lagoon of Tunis is very similar to that described for this species in the Venice Lagoon. Indeed Prevedelli et al. [15], showed that the gonadal activity was maximal during summer period in both sexes. Starting from November immature oocytes decreased and progressively those with a wider diameter increased in number. Spawning took place in April-May. We also noticed the presence of a few isolated mature males and females during the rest of the year indicating the presence of a sporadic reproduction or as stated by Prevedelli et al. [15], the fact that not all eggs are spawned, a small proportion being kept as a reserve material for the following gamete production. Partial oosorption and also total reproductive failure were observed in a wide variety of iteroparous marine organisms and have been interpreted as a component of a fitness response trade-off of “present reproduction” against “future reproduction” [23–25]. 

In the Woods Hole region, Pettibone [26] found that *M. sanguinea* laid eggs in masses of firm jelly in their burrows between June and July. So, the spawning period may change in different years or sites because gametogenesis and gamete release could be influenced by temperature, as in a number of polychaetes [23, 27–30]. In *M. sanguinea* the breeding season occurs in periods of the year where there are suitable conditions for larval development, as larvae do not tolerate low temperature [29].
